# Multiple malignancy-associated dermatomyositis myositis-specific autoantibodies in dermatomyositis

**DOI:** 10.1016/j.jdcr.2025.05.023

**Published:** 2025-06-16

**Authors:** Janet Choi, Adnan Mir, Benedict Wu

**Affiliations:** a Division of Dermatology, Department of Medicine, Albert Einstein College of Medicine/Montefiore Medical Center, Bronx, New York; b Dermpath Diagnostics, White Plains, New York

**Keywords:** dermatomyositis, Jo-1, malignancy, MDA-5, myositis-specific autoantibodies, NXP-2, SS-A-52, TIF1-γ, U1-RNP

*To the Editor:* We read with great interest Narayan and Richardson’s report of dermatomyositis (DM) with multiple myositis-specific autoantibodies (MSAs), including anti-transcriptional intermediary factor 1-γ (TIF1-γ), SUMO-activating enzyme, and nuclear helicase protein-2.^1^ Other known dermatomyositis-specific MSAs (DM-MSAs) include anti-nuclear matrix protein-2 (NXP-2) and melanoma differentiation-associated gene-5 (MDA-5). Multiple DM-MSAs are uncommon, with an estimated prevalence of 15.7% among MSA-positive patients.^2^ Herein, we present a DM patient with TIF1-γ, MDA-5, and NXP-2 autoantibodies.

A 30-year-old woman presented with a 1-month history of a heliotrope rash (Fig 1, *A*), Gottron sign (Fig 1, *B*), V-neck erythema (Fig 1, *C*), hypopigmented, red-on-white patches (Fig 1, *C* and *D*), and muscle weakness. Laboratory studies revealed elevated creatine kinase (11,484; normal range, 5-150 U/L) and aldolase (29.4; normal range, 3.3-10.3 U/L). The extranuclear antigen antibody panel showed antiribonucleoprotein positivity. The Myomarker 3 Panel was also positive for anti-histidyl-tRNA synthetase, TIF1-γ, MDA-5, NXP-2, U1-ribonucleoprotein, and Sjögren-syndrome-related antigen A 52 antibodies. A punch biopsy revealed perivascular and interstitial dermatitis with interface changes and pigment-laden macrophages (Fig 2, *A-C*). Malignancy and interstitial lung disease screenings were unremarkable.

**Fig 1:**
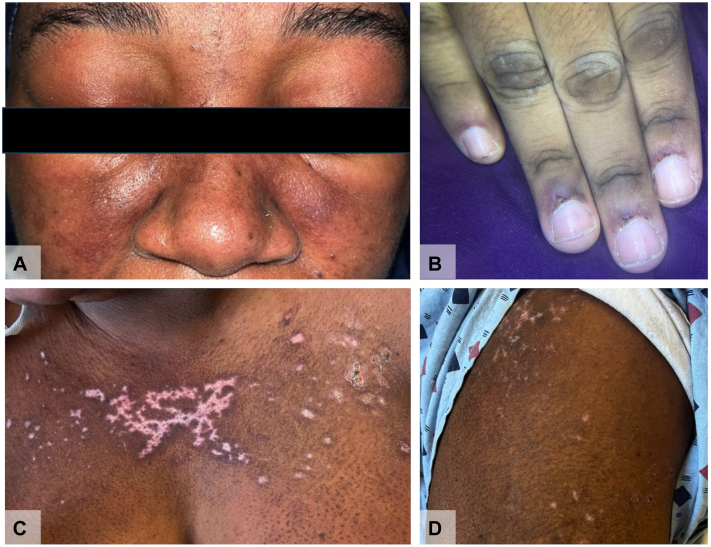
Clinical features of the DM patient with TIF1-γ, MDA-5, and NXP-2 autoantibodies, including (**A)** heliotrope rash, (**B)** Gottron sign on the distal and proximal interphalangeal joints with frayed cuticles and periungual telangiectasia, (**C)** V-neck erythema and dyspigmented plaque, and (**D)** hypopigmented and telangiectatic (“red-on-white”) patches. *DM*, Dermatomyositis; *MDA-5*, melanoma differentiation-associated gene-5; *NXP-2*, nuclear matrix protein-2; *TIF1-γ*, transcriptional intermediary factor 1-γ.

**Fig 2:**
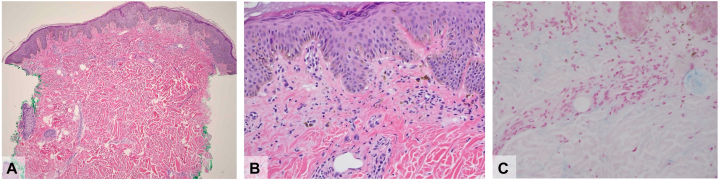
A punch biopsy from the back showed a sparse superficial perivascular lymphocytic infiltrate (**A)** H&E, ×4, with subtle hints of vacuolar degeneration and dermal melanin deposition (**B)** H&E, ×20. An Alcian blue stain highlighted foci of mucin deposition within the dermis (**C)** H&E, ×20. *H&E*, Hematoxylin and eosin.

Our case is notable for the presence of 6 distinct MSAs, including 3 DM-MSAs, 2 of which (NXP-2 and TIF1-γ) are associated with malignancy.^3^ The absence of vasculopathic ulcerations typically seen in the MDA-5 subtype and extensive hypopigmented and red-on-white telangiectatic patches suggests that our patient had the dominant TIF1-γ phenotype.^4^^,^^5^ This additional case of multiple positive DM-MSAs reinforces the importance of the clinical examination when evaluating DM patients.

## Conflicts of interest

None disclosed.
